# Medicinal and therapeutic properties of garlic, garlic essential oil, and garlic-based snack food: An updated review

**DOI:** 10.3389/fnut.2023.1120377

**Published:** 2023-02-16

**Authors:** Tarun Verma, Ankur Aggarwal, Priya Dey, Anil Kumar Chauhan, Summya Rashid, Kow-Tong Chen, Rohit Sharma

**Affiliations:** ^1^Department of Dairy Science and Food Technology, Institute of Agricultural Sciences, Banaras Hindu University, Varanasi, Uttar Pradesh, India; ^2^Department of Pharmacology and Toxicology, College of Pharmacy, Prince Sattam Bin Abdulaziz University, Al-Kharj, Saudi Arabia; ^3^Department of Occupational Medicine, Tainan Municipal Hospital (Managed by Show Chwan Medical Care Corporation), Tainan, Taiwan; ^4^Department of Public Health, College of Medicine, National Cheng Kung University, Tainan, Taiwan; ^5^Department of Rasa Shastra and Bhaishajya Kalpana, Faculty of Ayurveda, Institute of Medical Sciences, Banaras Hindu University, Varanasi, Uttar Pradesh, India

**Keywords:** garlic, antioxidant, organo-sulfur compounds, allicin, antimicrobial, essential oil

## Abstract

Garlic (*Allium sativum*) is an edible tuber belonging to the family Liliaceae. It has been used since ancient times as a spice to enhance the sensory characteristics of food and as a household remedy for the treatment of a variety of ailments. Garlic has been studied for its medicinal and therapeutic effects in the treatment of various human diseases for a long time. Health benefits associated with the consumption of garlic are attributed to the various sulfur compounds present in it such as allicin, ajoene, vinyl-dithiin, and other volatile organosulfur compounds which are all metabolized from alliin. Several researches in the literature have shown evidence that garlic exhibits antioxidant, antiviral, anti-microbial, anti-fungal, antihypertensive, anti-anemic, anti-hyperlipidemic, anticarcinogenic, antiaggregant, and immunomodulatory properties. The present review identifies and discusses the various health benefits associated with the consumption of garlic, its essential oil, and bioactive constituents, along with exploring the various snack-food products developed by incorporating garlic.

## 1. Introduction

Urbanization has brought many changes in society worldwide which includes consumers’ lifestyles and as a result their dietary practices. Snacks have become a quick and convenient food option for consumers. People are increasingly snacking in between main meals, originally to alleviate hunger but subsequently as a mainstream meal. According to Renub Research (1) latest report India’s snack food industry consists of many Indian as well as multinational companies. The market of snack food products in India is valued at US$ 11.08 Billion in 2020 and is expected to expand with a double-digit compound annual growth rate (CAGR) of 13.24% from 2020 to 2026 (1). Indian snacks market is categorized into Chips, Extruded Snacks, Namkeen, and others in which namkeen has the highest market value share in comparison to all other segments. Namkeen is currently the dominating category in both organized and unorganized markets in which fast-moving consumer goods (FMCG) companies capture a large market for its snacks segment. However, with the arrival of nutritional snacks enriched with organic ingredients with low calorie/oil content into the Indian market, these snacks are considered a bit healthier than conventional snacks and so are preferred by a large Indian population (1). Rising consumer health awareness is increasing the adoption of value-added product alternatives with natural, organic, low-calorie, vegan, gluten-free ingredients and components which imparts functional properties. With an increase in lifestyle ailments such as diabetes and cardiovascular disease, customers want to purchase products that are not only delicious and readily available, but nutritious as well.

Herbs and spices could be useful as a functional and flavoring ingredient as well as pharmacological and therapeutic properties in snack products (2). Garlic (*Allium sativum* L.; Liliaceae family) is one of the most important bulb crops which is grown and used as a spice and a popular Indian traditional medicinal plant (3). Garlic’s health benefits are mostly attributed to sulfur-containing components such as allicin, S-allyl cysteine and essential bioactive elements of garlic include organosulfur composites, thiosulfates and allicin (4, 5). Garlic paste and lime are used for mouth sore, sore throat and also can be used in toothpaste to prevent dental caries (6). It is reported that garlic is a potential unique therapeutic food, helpful to manage coronavirus disease (COVID-19) infection (7, 8). This comprehensive review encompasses the various medicinal and health beneficial roles by bioactive constituents associated with essential oil, and garlic-based snack-food products.

## 2. Nutritional composition of garlic

**FIGURE 1:**
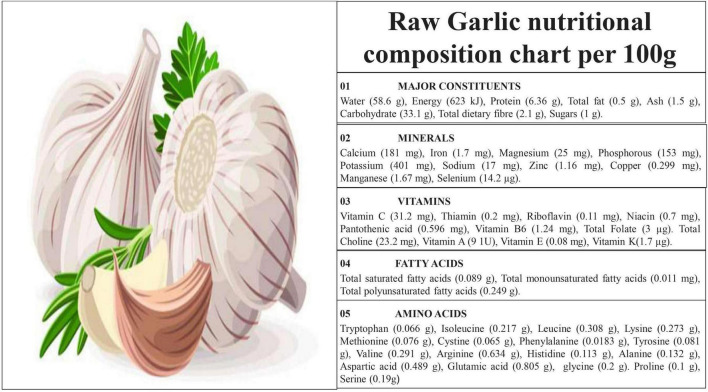
Nutritional composition of raw garlic per 100 g.

Garlic is a vegetable species that can be categorized as a food or a medicinal herb. It is a member of the *Amaryllidaceae* family or genus Allium that is cultivated all over the world. Various Nutritional composition of garlic per 100 g as per United States Department of Agriculture (USDA) 2019 is given in Figure 1 (9).

## 3. Bioactive components in garlic

**FIGURE 2:**
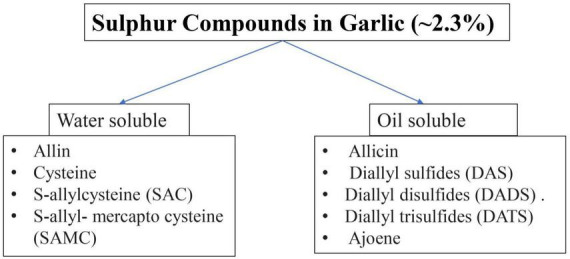
Sulfur compounds present in garlic.

Organosulfur compounds, saponins, phenolic compounds and polysaccharides are among the most common bioactive chemicals found in garlic (10). Onions are richer in protocatechuic acid than garlic, which has a high concentration of quercetin which is determined by high-performance liquid chromatography (11). Garlic bulb is reported to have total flavonoid (36.1 mg kg^–1^ FW), polyphenolic compounds (12.64–22.66 mg/1 g gallic acid), antioxidant activity (9.92–40.41 mol Trolox/g) evaluated using the DPPH technique (12–14). Organosulfur compounds and their derived products are primarily responsible for the bioactive characteristics of garlic, with diallyl thiosulfonate (allicin) having the major contribution. Other major organosulfur components are diallyl sulfide (DAS), diallyl disulfide (DADS), diallyl trisulfide (DATS), E-ajoene, Z-ajoene, S-allyl-cysteine (SAC), and S-allyl-cysteine sulfoxide (alliin) (15). Various sulfur components found in garlic is given below in Figure 2 (16).

## 4. Doses as per the WHO

Daily dose of garlic for adults as per suggested by World Health Organization (WHO) is listed below in Table 1.

**TABLE 1:** Various garlic product and their daily dose (17).

Garlic product	Dose/Day
Fresh raw garlic	2–5 g
Dried garlic powder	0.4–1.2 g
Garlic oil	2–5 mg
Garlic extract (solid)	300–1,000 mg
Aged garlic extract (liquid)	2,400 mg

## 5. Types of garlic products available in the market

**FIGURE 3:**
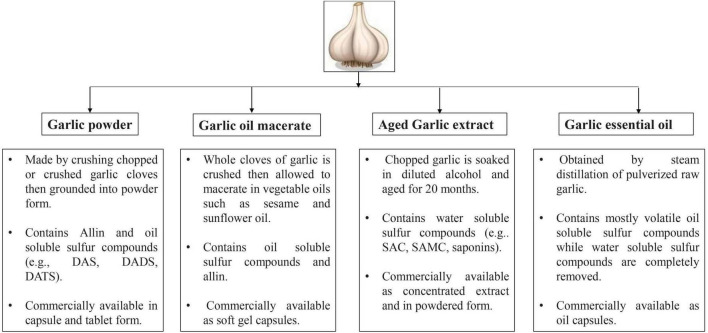
Various types of garlic products available in market.

Garlic products can be categorized under different categories like garlic powder, garlic oil macerate, aged garlic extract and garlic essential oil. Description of various garlic products are elaborated in the Figure 3.

## 6. Chemical changes in garlic after processing

**FIGURE 4:**
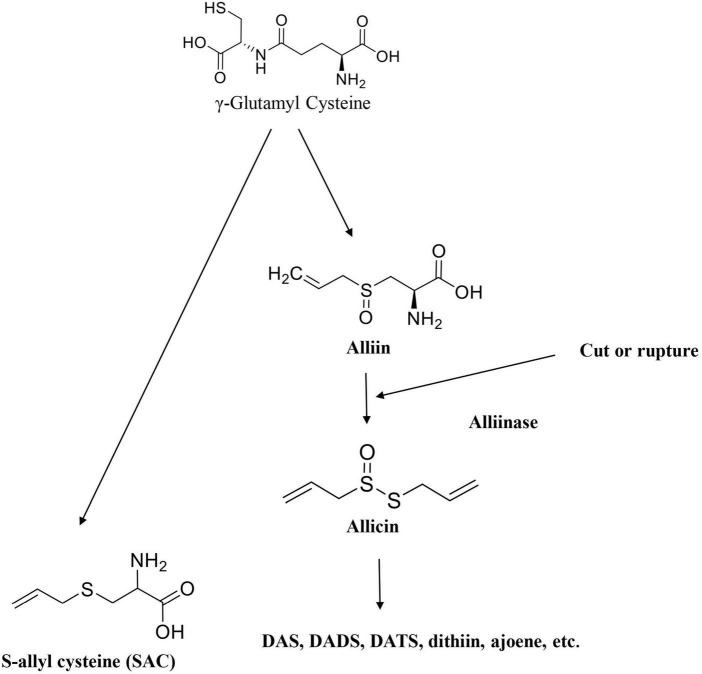
Various bioactive components produced by garlic when subjected to processing.

Raw garlic bulbs have an abundance of γ-glutamyl-cysteine in intact form. Alliin forms naturally from these components when stored under low temperatures. The enzyme alliinase breakdown alliin to create thiosulfinates like allicin after the garlic has undergone processing operations like chopping, chewing or crushing, or any other operations that disrupt the cell membrane. Allicin and other thiosulfinates breakdowns into DAS, diallyl trisulfide (DAT), DADS, dithiins, and ajoene very instantaneously while simultaneously γ-glutamyl-cysteine is converted to SAC by a different mechanism (18). Thiosulfinates, especially alliin is the most common precursor responsible for garlic’s flavor and these sulfur compounds are also responsible for garlic’s well-known therapeutic properties (19). Various Bioactive components produced when garlic undergoes processing are given in Figure 4.

## 7. Health benefits of garlic

Have studied the effects of garlic consumption on decreasing total cholesterol (TC) and low-density lipoprotein (LDL) is more pronounced with a lower dosage and longer duration, especially in individuals with cardiovascular diseases (20). Raw garlic and garlic extract in the form of oil or powder can be utilized as functional and therapeutic food. There is significant evidence that indicates preventive and therapeutic roles of garlic in improving the immune system, anti-tumor properties and antioxidant activity of garlic protects the body against free radicals (21, 22). Human health has been found to benefit from a balanced diet rich in functional foods prepared with garlic. Garlic can alter blood anticoagulant levels and boost the activity of various organs in the body mainly of respiratory and digestive systems (23). According to evidence from preclinical investigations and clinical trials, garlic consumption appears to have a significant impact on antihypertensive (24), antidiabetic (25), immune-modulatory (26) and hypolipidemic effects (27) and it would be highly beneficial for medical and surgical treatments (28). Garlic is reported to lower the amount of the gastrointestinal illness-causing cryptosporidiosis in immunocompromised mice and reduced inflammation (29). Garlic alone can give us over 200 unique chemicals that can help in strengthening the immune system and help fight the body against a range of ailments. Garlic’s bioactive components can be protected using encapsulation techniques (30). Under biotic and abiotic stress circumstances, garlic extract has been demonstrated to enhance crop quality and soil conditions (31). When compared to neem oil, clove oil, and tulsi oil, garlic oil is more efficient against aerobic bacteria (32). A list of health benefits associated with various types of garlic products, along with their bioactive components is given in Table 2.

**TABLE 2:** List of health benefits associated with different types of garlic product along with its bioactive components.

Garlic product	Bioactive components	Health benefits	Area of research	References
Aged garlic extracts	Sulfur, saponins	Immune modulatory (IM) and anti-inflammatory effects, cells protection against oxidative stress	IM and anti-inflammatory effects mainly due to its various sulfur-containing components.	(33, 34)
2.5-gram fresh garlic per day	Allicin	Alleviating osteoarthritis	The garlic group dropped serum levels c-reactive protein (CRP) and tumor necrosis factor-alpha (TNF- α) in comparison to the placebo group in obese or overweight women.	(35)
Black garlic extract	Diallyl sulfide, Diallyl disulfide	Stimulates the muscles in the digestive tract which aids in the passage of food and promotes bowel movement.	Black garlic *n*-butanol fraction increased 5-Hydroxytryptamine receptor concentration which efficiently promotes gastrointestinal tract movement.	(36, 37)
Garlic “Rosato” and “Caposele”	Allicin	Inhibiting the growth of bacteria	Rosato extract was more efficient in antimicrobial activity i.e., *Staphylococcus aureus*, *E. coli*, *Pseudomonas aeruginosa*, *Penicillium expansum*, and *Bacillus cereus.* Caposele extract was more efficient at preventing growth in *Aspergillus versicolor* and *Penicillum citrinum.*	(38)
Raw garlic, garlic oil	Allicin	Inhibiting growth of bacteria in the stomach	Garlic showed significant antibacterial activity i.e., *Helicobacter pylori* infection in stomach, which was evaluated by urease breath test.	(39)
Crude garlic extract	Bioactive lipid compounds	Induces apoptosis of bacterial cell	Crude garlic extract inhibits the growth of cancer cells and causes cell cycle arrest and apoptosis.	(40)
Garlic	Antioxidant	Blastocystosis	Blastocystis cyst shedding was reduced significantly in comparison to the untreated infected group.	(41)
Garlic extract	Antioxidant, phenolic compounds	Antimicrobial activity against pathogenic microorganisms	Garlic was found to be effective against pathogenic micro-organisms such as *Escherichia coli* and *Pseudomonas aeruginosa.*	(42)
Garlic outer skin	Phenolic compound	Bacterial growth inhibition	Garlic husk waste acts as a potential source of phenolic compounds i.e., trans-N-feruloyltyramine, luteolin, luteolin-7-O-β-Glucopyranoside, apigenin, and chrysoeriol compounds which may act as antimicrobial activity.	(43)

### 7.1. Anti-viral properties

Garlic and its oligosaccharides have been proven in preclinical testing to exhibit antiviral properties (44). Demonstrated the antiviral effects of aqueous Garlic extracts against coronavirus (co-treatment and post-treatment). Garlic extracts have been proven to be effective against embryonic eggs that are infected with the coronavirus in aqueous form, suggesting that they may stop or lessen viral proliferation (45). Garlic has been used to cure a variety of infections in Africa, including sexually transmitted diseases, tuberculosis, wounds, and infections of lungs (46). High organosulfur compounds in garlic essential oil are reported to interact strongly with the amino acids of the angiotensin-converting enzyme (ACE2) protein which prevent COVID-19 and the PDB6LU7 protein (main protease of SARS-CoV-2) (47). Garlic has been studied in a huge number of preclinical antiviral investigations against viruses with effective results in Table 3.

**TABLE 3:** Effect of garlic’s various Organo Sulfur Compounds (OSCs) and its extract on various types of viruses.

Name of virus	Common infection	Area of research	References
Coronavirus (CoV), severe acute respiratory syndrome coronavirus (SARS-CoV)	Respiratory tract infection (animal and human)	Garlic extracts affect the replication of influenza A virus during the early stages of infection.	(48)
Porcine Rotavirus (PRV); Rotavirus SA-11 (RV-SA-11)	Gastrointestinal infection and diarrhea in animal and human	Garlic’s ethnomedicinal use in diverse viral infections have been confirmed (*in-vitro* and *in-vivo* pharmacological tests) in distinct garlic extracts.	(49, 50)
Potato Virus Y (PVY)	Virus infecting potato	Red chili (*Capsicum annum* L.), garlic, neem (Azadirachta indica A. Juss.) and pyrethrum flower (*Chrysanthemum* sp.) extracts, used single or combination offer some aphid control especially in the early stages of infestation.	(51)
Human papillomavirus (HPV)	Refractory multiple common warts (RMCW)	Research found that 10% garlic extract has effect on male genital warts that was comparable to cryotherapy treatment.	(52)
Coronavirus disease (COVID-19)	Virus infecting	Individuals with mild to moderate symptoms of COVID-19 were given garlic essential oil as compared to those patients who were not given the essential oil.	(53)

### 7.2. Anti-microbial properties

Garlic contains compounds that can prevent bacterial proliferation or cause apoptosis without harming the infected organism. To fight these microorganisms, garlic is considered as strong as broad-spectrum antibiotics (54). Discovered that chloroform extract of aged and non-aged garlic extract had a remarkable antimicrobial activity against *Staphylococcus aureus, Salmonella enterica, E. coli (Escherichia coli)*, and *Listeria monocytogenes*. Garlic is reported to be more effective with fewer side effects than commercial antibiotics; as a result, it may be utilized as a substitute for antibiotics (55). Garlic has recently been found to exhibit antibacterial properties against a wide spectrum of gram-negative bacteria such as *Aeromonas hydrophila* (56), *Pseudomonas aeruginosa* (57), *E. coli* (58), and gram-positive bacteria such as *Bacillus cereus* (54), *Streptococcus mutans* (59), *Staphylococcus epidermidis* (60) and Methicillin-resistant *Staphylococcus aureus* (61). Garlic extracts have a broad antibacterial spectrum that includes gram-negative and gram-positive microorganisms (62). Garlic extracts demonstrate antibacterial activity due to various bioactive components present in them which breaks down bacterial cell membrane resulting in bacterial cell death (63).

### 7.3. Anti-fungal properties

Garlic extracts were found to possess broad-spectrum fungicidal activity against *Candida* (64, 65), *Trichophyton* (66), *Aspergillus* (65) and *Rhodotorula* spp (67). Garlic extract which acts as antifungal (68) and prevent the growth *Meyerozyma guilliermondii* and *Rhodotorula mucilaginosa* (69). Additionally, garlic oil can be applied externally to cure ringworm, warts, and skin parasites (70). A study carried out synthesis of environment-friendly silver nanoparticles using hill garlic extract with enhanced antifungal properties (71). Garlic oil mainly include ingredients *viz*. disulfides (36%), trisulfides (32%) and monosulfides (29%) which have strong antifungal activity i.e., *Penicillium funiculosum* (47).

### 7.4. Anti-tumor properties

Garlic extract ability to inhibit cancer growth activity *in vivo* against bladder cancer prevention (72). Garlic’s ability to inhibit cancer growth i.e., tumor vessel formation by inducing the expression of survival genes work based on stimulating helper T-cells which is linked to helping the immunity system fight various infections as well as cancer (73). Garlic showed strong anti-cancer activity, particularly in connection to digestive tract tumors. Consumption of garlic reduced the risk of esophagus, stomach, and colon cancer, according to human population research (74). Several bioactive compounds in garlic including DATS, allicin, DADS, diallyl sulfide, and allyl mercaptan have anticancer properties (75).

### 7.5. Anti-oxidant properties

Garlic’s nutritional and phenolic compounds have excellent antioxidant status in cancer, post-prandial lipemia patients, reducing oxidative stress (76, 77). Garlic’s antioxidant effect is due to its ability to modulate ROS while also raising glutathione and cellular antioxidant enzymes (78). It has been shown that aged garlic extracts (AGE) are effective against atherosclerosis as it helps in the reduction of reactive oxygen species (ROS) and thus prevent endothelial activation and dysfunction (79).

### 7.6. Cardiovascular protection

**FIGURE 5:**
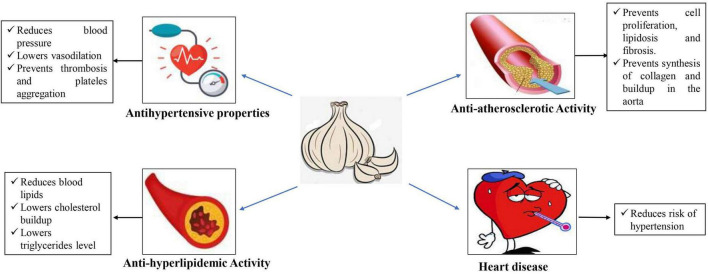
Effects of garlic on cardiovascular protection.

Garlic can lower blood lipids, decrease CVD risk factors, and improve HDL levels in addition to enhancing cardiovascular parameters such microcirculation, epicardial and perivascular adipose tissue, post occlusive reactive hyperemia, and carotid artery intima media thickness (75). Garlic has the potential for reducing cardiovascular diseases since it lowers both systolic and diastolic blood pressure (80). It is a well-established fact that Garlic is having many cardiovascular protection properties as shown in Figure 5.

#### 7.6.1. Anti-hypertensive properties

The antihypertensive action of garlic is mainly due to organosulfur compounds, which promote factors that relax endothelium and lowering blood pressure (81). Garlic extracts have been shown to be effective in aqueous form against coronavirus-infected embryonic eggs, suggesting that they may prevent or reduce viral growth (82). Furthermore, garlic was found to be effective in preventing thrombosis and platelet adhesion or aggregation in people (83).

#### 7.6.2. Anti-hyperlipidemic activity

Processing 1.5% black garlic lowered total cholesterol, alter triglyceride and low-density lipoprotein cholesterol in rats fed a diet which having high in cholesterol (84, 85). Aged garlic extract (AGE) was found to lower the blood pressure by 3.75 mm Hg systolic and 3.39 mm Hg diastolic, whereas garlic supplements lowered blood pressure by about 10 mm Hg systolic and 8 mm Hg diastolic, similar to that of conventional BP medication (86).

#### 7.6.3. Anti-atherosclerotic activity

Dyslipidemia and inflammation are major indications of atherosclerosis, a chronic disease, which develops depending on several factors (87). Atherosclerosis builds up over time in human arteries as plaque and might go unnoticed for a long period (88). Garlic preparation has direct anti-atherogenic action and inhibits the development of cholesterol-induced experimental atherosclerosis (89). Garlic extracts inhibited sialidase activity in blood plasma, which is the major cause of the formation of atherogenic low-density lipoprotein (90).

#### 7.6.4. Heart disease

Heart disease is considered to be one of the biggest causes of death worldwide, which includes heart-related diseases like hypertension. Studies have shown that garlic can help people who are suffering from hypertension by lowering their blood pressure (91). By partially increasing Na+/K+-ATPase levels, garlic and its identified metabolites can inhibit iso-induced hypertrophic development in rat heart tissue and H9C2 cell lines (92). In people with moderate hypercholesterolemia, aged black garlic (AGD) extract with a standardized SAC yield in conjunction with dietary suggestions about cardiovascular diseases (CVD) risk factors (93). Garlic and garlic include selenium (Se) has also been shown to reduce blood cholesterol, which is a key factor in causing heart disease (94, 95).

### 7.7. Anti-diabetic properties

Diabetes, often known as diabetes mellitus, is a fatal metabolic disorder characterized by high blood sugar levels mainly due to the body either cannot use insulin effectively or does not produce enough of it (96). Preclinical research demonstrated that garlic’s active sulfur-containing compounds lowered hyperglycemia by enhancing the antioxidant capacity in diabetic rat circulatory systems (97). Furthermore, the garlic component functions as a donor of hydrogen sulfide, which regulates type 2 diabetes (98). Garlic (300 mg garlic two times per day for 12 weeks) significantly improved blood triglycerides, LDL and lower glucose parameters (99). Furthermore, compared to placebo diabetic patients with uncontrolled dyslipidemia, decreased serum lipid levels.

### 7.8. Anti-rheumatic properties

Osteoarthritis (OA), is a degenerative disease of the bone joints that causes chronic and severe pain (35). After 12 weeks of treatment, a garlic supplementation of 1,000 mg per day was found to be useful in the alleviation of symptoms in obese women suffering from osteoarthritis in the knees. Furthermore, garlic tablets (500 mg twice a day for 12 weeks) have anti-inflammatory and painkiller effects, lowering serum resistance and TNF- α levels as well as pain intensity in obese women with osteoarthritis of the knee (35). Garlic tablet acts as an antioxidant in postmenopausal women suffering from osteoporosis, and reduction in oxidative stress, according to a randomized clinical study (100).

## 8. Effect on male fertility

Garlic contains sulfur compounds influence the activity of the enzyme family’s glutathione S-transferase (GST) which is known to detoxify carcinogens and cytochrome P450 (CYP), which is known to activate a variety of chemical carcinogens in test animals (101). Moreover, the Antioxidant activity of garlic can help with fertility by lowering the peroxidation of lipids (102). Research has also found that the antioxidant effects of garlic can minimize the toxicity of damaging medications on the testes while also increasing spermatogenesis and fertility in men (103). Garlic possesses phytoestrogens, which have a direct influence on estrogen (104). Research has revealed that ROS which linked to the development of male infertility, plays a critical role in the disruption of the spermatogenesis process i.e., diminished ability of the genital system’s and sperm’s antioxidant system (105, 106). Diallyl disulfide present in garlic, protects the sexual organs by reducing ROS, improving and strengthening the blood-testis barrier, and increasing blood flow in the testicles (107).

## 9. Mechanism of action of phyto-constituents in garlic

**FIGURE 6:**
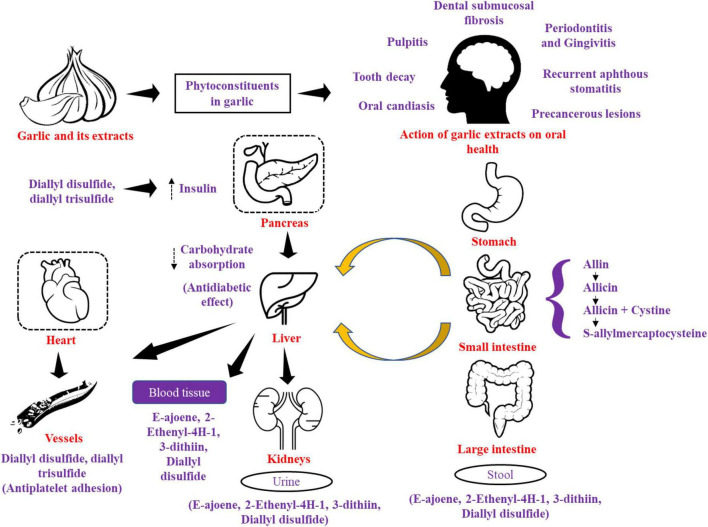
Mechanism of action of various phytoconstituents in garlic as it passes through gastrointestinal tract.

Allicin and its metabolites can be identified in the blood, feces, and urine after ingesting 25 g of raw garlic which is responsible for most of the therapeutic activity and it is converted to allyl-mercaptan almost immediately under an enzyme-inhibiting gastrointestinal environment (108). According to studies conducted on raw garlic and preparation products, the primary sulfur-containing groups display varying bioavailability between raw garlic and various garlic formulations like water-soluble organo-sulfur compound SAMC has anti-oxidant properties which inhibits cell proliferation, boosts apoptosis in cancer cell lines (109) and SAC which has a blockage of nitrosamine generation and bioactive whereas oil soluble organo-sulfur compound i.e., allicin has suppressed the proliferation of cancer cells, increased apoptosis by activation of caspases and diallyl-disulfide inhibit Colon cancer by suppressing the growth of neoplastic Canine Mammary Tumor cell (CMT-13) and N acetyltransferase (10, 110, 111). The higher bioavailability of allicin (the main bioactive compound of garlic), allyl methyl sulfide in garlic-based foods as compared to crushed raw garlic (112) and higher bioavailability of allicin in enteric tablets was more than in garlic capsules. At body temperature cystine which is released from protein diet interacts with allicin in gastrointestinal tract (GI) tract to generate two S-allylmercaptocysteine (SAMC) derivatives (113). Allicin’s secondary metabolites, such as E ajoene, 2-ethenyl-4-H1, 3-dithiin, and DADS, can be found in the blood and urine after the metabolism of allicin (114). The mechanism of action of various phytoconstituents in garlic as it passes through the gastrointestinal tract is given in Figure 6.

## 10. Garlic essential oils: Their composition and properties

**FIGURE 7:**
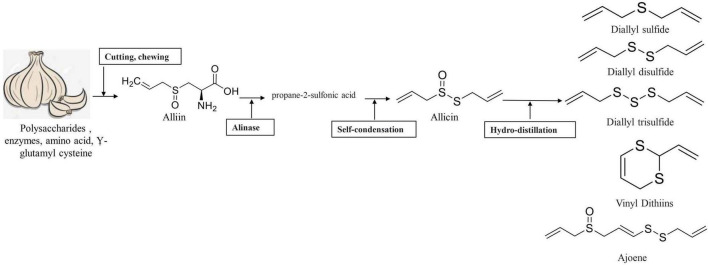
Conversion of allicin into the main components of garlic essential oil.

Garlic which has medicinally chemicals compounds primarily γ-l-glutamyl-l-cysteine, is found in mature garlic bulbs (115). The amino acid alliin, which is an alkyl derivative of cysteine alkyl sulfoxide, is the major component in whole garlic bulbs. The enzyme alliinase is released when tissues are crushed, chewing that converts cytosolic cysteine sulfoxides (alliin) into thiosulfinates (Figure 7). These substances are smelly, volatile and reactive (18, 116).

**FIGURE 8:**
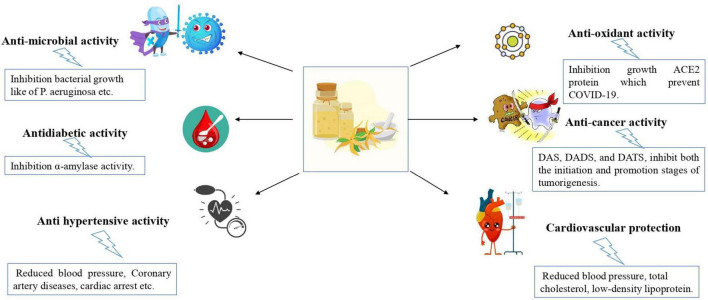
Biological activity of garlic essential oil.

Garlic oil (GO) which is utilized in many medical garlic products, is created by steam distilling mashed garlic and creating organo-sulfur compounds and yields between 0.09 and 0.35 % of the fresh weight (117). Tocmo et al. (118) assert that DATS is more prevalent in fresh GO than commercial oils, with the number of DADS varying depending on the temperature or duration of the extraction process. The amount and number of constituents in essential oils vary, but in all investigations reported diallyl disulfide is almost always the main ingredient followed by allyl disulfide, allyl trisulfide (119). These substances are the transformation products from allicin, a substance with significant medicinal value because it has a wide range of biological activities. However, allicin is also the most unstable of all the thiosulfinates produced by the vascular enzyme allinase, which is released after tissue damage (120). GO is particularly a good source of organosulfur compounds, primarily allyl disulfide (28.4%), allyl trisulfide (22.8%), allyl-1-propenyl disulfide (8.2%), allyl methyl trisulfide (6.7%), and diallyl tetrasulfide (6.5%) (121). These compounds have potent antioxidant, antibacterial, antithrombotic, and immunomodulatory properties as well as hypotension, anticancer and antimicrobial effects (121) (Figure 8).

### 10.1. Antimicrobial

The antimicrobial action of undiluted form of GO is 900 times more potent than fresh garlic and 200 times more potent than garlic powder (122). Another allicin-derived molecule called allyl methyl sulfide is reasonably stable in blood, suggesting that the use of GO in the treatment of numerous infectious disorders (123). According to Li et al. (124), diallyl disulfide a significant organo-sulfur component of GO inhibits the transcription of *P. aeruginosa’s* important genes to suppress the growth of virulence factors. In the food industry, fresh GO is utilized as a natural antioxidant, flavoring ingredient, and antibacterial, especially in gram-negative bacteria like *Escherichia coli* and *Pseudomonas aeruginosa* in processed chicken and meat products (125). The antifungal effectiveness of tomato plant Alternaria leaf spot disease produced by essential oil-encapsulated lipid nanoemulsions (126). Natural ingredients i.e., rosemary essential oils and GO which limited the growth of aerobic microorganisms, S. *aureus, Salmonella* spp., *B. thermosphacta*, molds and yeasts, lactic acid bacteria and coliform that act natural preserve meat and meat products (127).

### 10.2. Antioxidant

The antioxidant capacity of wet and dry heated garlic (70, 100, and 121°C) was investigated and it was reported that heating diminishes antioxidant potential due to the breakdown of phenolics and sulfur-containing compounds (128, 129) found that even at a dose of 200 mg of GO demonstrated strong antioxidant activity which was comparable to vitamin C.

### 10.3. Cardiovascular

According to earlier research, consuming garlic power reduced blood pressure, total cholesterol, low-density lipoprotein, and other risk factors that could lead to cardiovascular disorders (130). In clinical trials, GO has exhibited significant applications in CVDs including intracellular calcium overload, oxidative stress, inflammation, vascular endothelial cell injury and dysfunction, and dyslipidemia (131).

### 10.4. Antihypertensive

According to data, people who consume more garlic are more likely to have low blood pressure (132). According to epidemiological research, there is a correlation between garlic consumption and hypertension in rats, which lowers the risk of cardiovascular problems (133). Biological activities of GOs such as angiotensin-converting enzymes which have inhibitory potential, α-amylase and α-glucosidase which have inhibition potential antihypertensive activity, and antidiabetic activity (25).

### 10.5. Anti-cancer

It has been shown that the components of GO which contain diallyl sulfide and diallyl disulfide compound prevented mutagenesis by blocking cytochrome P-450 2E1, which is required for the conversion of the cancer cells (134). When treating cancer, essential oils (EOs) can be combined with synthetic drugs which can boost immunity (135). Greater celandine (*Chelidonium majus* L.) essential oil-infused chitosan nanoparticles as an anticancer agent on the MCF-7 cell line (136). In experimental carcinogenesis models for several forms of cancer, the major components of GO, such as DAS, DADS, and DATS, were found to inhibit both the initiation and promotion stages of tumorigenesis (137).

### 10.6. Antidiabetic

In one clinical trial, Zhang et al. (134) gave gelatin capsules containing 8.2 mg of GO daily to the test group for 11 weeks and showed that chronic GO consumption significantly reduced blood sugar levels in the treated female portion of the group, while men group displayed an increase in blood sugar levels. A study revealed that there are gender disparities in the benefits of garlic and healthy men must take more GO supplements in order to have the desired effects on blood sugar and cardiovascular health (138).

## 11. Possible toxicities of garlic and garlic essential oil

The U.S. Food and Drug Administration has classified garlic as “Generally regarded as safe (GRAS)” for food and flavoring ingredients, although there are severe consequences of reported acute and chronic toxicity on its excessive consumption. Allicin, a substance abundant in garlic, when consumed in large doses, can be hazardous to the liver (139). Additionally, the abundance of different sulfuryl derivatives in essential oils may worsen its harmful effect such as throat and mouth burning, stomach ulcers, nausea, vomiting, erythematous mucosa which is characterized by redness and inflammation in the gas mucosa layer as well as hyphaemia, bleeding gums and potentially irreversible eyesight loss (121). The organosulfur compounds having possible toxicity i.e., endotoxin-induced systemic inflammation and intestinal damage is reported by a study (140).

## 12. Industrial application

Garlic is used in both cooking and medicine. Compelling evidence reports its possess anti-inflammatory, anti-hypertensive, anti-anemic, anti-hyperlipidemic, anticarcinogenic, antiaggregant, antioxidant, antiviral, anti-microbial, anti-fungal, and antiviral activities. Increased shelf life of all examined goods was enhanced by using fresh garlic and ready-to-eat garlic products with antioxidant properties that reduced the amount of free radical-scavenging activity and increase total polyphenol content (141). These beneficial components in garlic could be used to great success in the creation of foods and nutritional supplements for young children, pregnant or nursing women, cancer and cardiovascular death rates, as well as severe side events and morbidity after garlic therapy.

### 12.1. Garlic-based snack foods

Various garlic-based snack products have been developed as a result of studies which are given in Table 4. Commercially available garlic-based snack foods are given in Table 5.

**TABLE 4:** Research and development of garlic-based snack products.

Product	Main ingredients	Area of research	References
Whole grain gluten-free high protein buckwheat-kale snacks	Base ingredients- Buckwheat flour+ Peanut meal+ Kale (BPK) and garlic	Sensory acceptance: BPK-Garlic = 94% acceptance, BPK-Onion = 86% acceptance, BPK-Ginger = 78% acceptance.	(142)
Whole grain gluten-free vegetable spicy snacks	Base ingredients- Brown rice flour, Sorghum flour, Tapioca flour, Mashed potato, Canola oil, Guar gum, Baking powder and salt. Formulations- 1. Garlic (5%) and Carrot. 2. Garlic (5%) and Broccoli. 3. Garlic (5%) and Spinach.	Sensory acceptance: Carrot-Garlic = 77% acceptance Broccoli-Garlic = 68% acceptance, Spinach-Garlic = 61% acceptance, and Red Onion = 53% acceptance.	(143)
Garlic incorporated ready-to-eat extruded snacks	Garlic (upto 10%), rice, defatted soy flour	Garlic powder (5, 10%) in ready-to-eat extruded snacks with good acceptability and it can be preserved for 2 months using nitrogen packing as an effective packaging method.	(144)
Garlic and pepper-flavored puffed snacks	Salt (1.1%), garlic powder concentration (2.8%), and pepper powder (0.73%.)	A daily intake of 25 gram of the puffed snack would suffice to achieve the necessary daily garlic intake.	(145)

**TABLE 5:** Some commercially available garlic-based snacks.

Product name (brand name)	Product description	Visual	Nutritional composition/100 g
Butter garlic naan chips (wingreens farms)	Refined wheat flour, whole wheat flour, malted barley flour, millet flour, garlic, onion and other ingredients	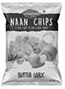	Calories (Kcal)—446 Total fat—14.8 g Saturated fat—5.9 g Total carbohydrate—68.1 g Sugars—2.3 g Protein—10.2 g
Garlic murukku (Telugu Foods)	Rice flour (64%), refined peanut oil, chickpea flour, garlic paste, and other ingredients	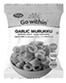	Energy (Kcal)—533 Total fat—32 g Saturated fat—0 g Total carbohydrate—60 g Sugars—0 g Protein—2 g Sodium—214 mg
Chili garlic khakhra (Jabsons chili)	Whole wheat flour (74%), garlic powder (3%), and other ingredients	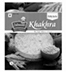	Energy (Kcal)—502 Total fat—23.7 g Saturated fat—6.4 g Trans fat—0.0 g Total carbohydrate—63.7 g Dietary fibre—3.0 g Sugars—4.7 g Protein—8.6 g Sodium—1,212 mg
Herb garlic toasties (the flavor republic)	Refined wheat flour, butter, garlic, and other ingredients	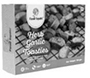	Energy (Kcal)—435.84 Total fat—17.36 g Saturated fat—10.07 g Cholesterol—Not detected Total carbohydrate—64.58 g Dietary fibre—3.0 g Sugars—3.15 g Protein—5.32 g
Low fat garlic toast with herbs (neelam foodland)	Wheat flour, garlic, and other ingredients	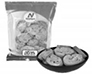	Energy (Kcal)—442 Total fat—13.18 g Total carbohydrate—71.47 g Sugars—6.20 g Protein—9.45 g
Special garlic chakli (neelam foodland)	Rice flour, gram dal, moong dal and garlic paste, and other ingredients	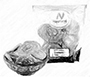	Energy (Kcal)—455 Total fat—19.6 g Total carbohydrate—56.6 g Sugars—0.0 g Protein—12.4 g
Wholesome roasted chilly garlic peanuts (Healthysthan)	Roasted peanuts, garlic powder, mango powder, and other ingredients	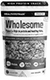	Energy (Kcal)—645 Total fat—52.31 g Total carbohydrate—20.17 g Dietary fiber—1.16 g Sugars (natural)—1.12 g Protein—23.56 g
Roasted chili garlic sticks (Agri club)	Whole wheat flour, garlic, red chili powder, and other ingredients	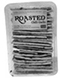	Energy (Kcal)—401 Total fat—13.37 g Saturated fat—2.17 g Cholesterol—0.0 mg Total carbohydrate—59.68 g Dietary fiber—4.06 g Sugars—0.0 g Protein—10.58 g
Roasted makhana chilly garlic (eatier)	Roasted makhana, spices mix, garlic powder, and other ingredients	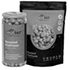	Energy (Kcal)—490 Total fat—22 g Total carbohydrate—65.5 g Sugars—10 g Protein—9 g
Garlic bhujia snacks (let’s try)	Gram pulse flour, garlic paste, corn flour, pure groundnut oil, and other ingredients	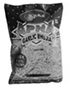	Energy (Kcal)—506 Total fat—26.4 g Trans fat—0.1 g Total carbohydrate—49.7 g Protein—9.5 g
Kalewa crisp namkeen (Kalewa)	Gram pulse flour, edible vegetable oil (soyabean, palmolin), garlic, spices and condiments, and other ingredients	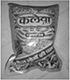	Energy (Kcal)—619 Total fat—40 g Trans fat—0 g Cholesterol—0.0 mg Total carbohydrate—20 g Sugars—0.0 g Protein—20 g
Garlic sev (yuvraj food product)	Moth flour, chana flour, garlic, and other ingredients	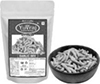	Energy (Kcal)—535.2 Total fat—40 g Saturated fat—10.00 g Trans fat—0 g Total carbohydrate—34.08 g Sugars—1.02 g Protein—9 g Sodium—746 mg

## 13. Herb-drug interactions

The possibility of interactions between herbal medicines and conventional pharmaceuticals is a significant safety concern with the increased usage of herbs. Herb-drug interactions have the same mechanisms as drug-drug interactions and can act both pharmacokinetic (changes in plasma drug concentration) and pharmacodynamic (drugs interact at receptors on target organs) levels (146). The induction (or inhibition) of hepatic and intestinal drug-metabolizing enzymes, specifically cytochrome P450 (CYP) and/or drug transporters like P-glycoprotein, as well as pharmacokinetic interactions, which have been more thoroughly studied, maybe the cause of the altered drug concentrations caused by co-administration of herbs (147). Clinical research indicates that ingesting garlic may cause pharmacodynamic interactions that could pose a risk to individuals on conventional medications, especially in those taking antihypertensive, anticoagulant, or hypoglycemic medications. Garlic interacts with antihypertensive medications that are prescribed to control blood pressure. Additionally, it interacts with Saquinavir and low therapeutic properties the amount of medication in the blood and circulatory system (148). Garlic enhances the effects of drugs that decrease cholesterol and lower blood pressure in the body (149). Garlic’s impact on hypoglycemic medications increases the likelihood of hypoglycemia, among other drug interactions (150). Anticoagulants including heparin, warfarin, and aspirin interact with the garlic plant to increase the risk of bleeding (149). Additionally, garlic increases the fibrinolytic and platelet-activating anti-factor activities in anesthesia (148).

## 14. Conclusion

Garlic is a aromatic herbaceous plant that is used as an essential ingredient in ready-to-eat snacks foods to enhance the flavor of the product and also improve the therapeutic, functional value and shelf life. Additionally, garlic is rich in organosulfur and flavonoid compounds which are effective in the management of wide range of health disorders, and may be a useful remedy in the prevention of COVID-19. Snacks can be nutritionally enriched by incorporating garlic extracts. The multifunctional therapeutic role of Garlic is attributed to its rich phytocompounds and their several bioactive properties including anti-microbial, anti-inflammatory, anti-hypertensive, anticarcinogenic, antifungal, antiviral, and antioxidant. More standard experiments and clinical studies are needed to back up the claims of garlic in the treatment and prevention of various diseases.

## Author contributions

TV, PD, AA, and RS: conceptualization, methodology, investigation, data collection, and writing—original manuscript. SR, K-TC, AC, and RS: editing and proofreading. RS and TV: supervision. All authors approved submission of the final manuscript.
